# A Triple Amino Acid Substitution at Position 88/94/95 in Glycoprotein GP2a of Type 1 Porcine Reproductive and Respiratory Syndrome Virus (PRRSV1) Is Responsible for Adaptation to MARC-145 Cells

**DOI:** 10.3390/v11010036

**Published:** 2019-01-08

**Authors:** Jiexiong Xie, Ivan Trus, Dayoung Oh, Lise K. Kvisgaard, Julie C. F. Rappe, Nicolas Ruggli, Nathalie Vanderheijden, Lars E. Larsen, François Lefèvre, Hans J. Nauwynck

**Affiliations:** 1 Laboratory of Virology, Faculty of Veterinary Medicine, Ghent University, Salisburylaan 133, B-9820 Merelbeke, Belgium; jiexiong.xie@ugent.be (J.X.); Ivan.Trus@gmail.com (I.T.); Dayoung.oh@ugent.be (D.O.); Nathalie.Vanderheijden@ugent.be (N.V.); 2 National Veterinary Institute, Technical University of Denmark, 2800 Lyngby, Denmark; likik@vet.dtu.dk (L.K.K.); lael@vet.dtu.dk (L.E.L.); 3 The Institute of Virology and Immunology IVI, 3147 Mittelhäusern and Bern, Switzerland; julie.rappe@crick.ac.uk (J.C.F.R.); Nicolas.Ruggli@ivi.admin.ch (N.R.); 4 Department of Infectious Diseases and Pathobiology, University of Bern, 3012 Bern, Switzerland; 5 Graduate School for Cellular and Biomedical Sciences, University of Bern, 3012 Bern, Switzerland; 6 INRA, Molecular Immunology and Virology Unit, 78350 Jouy-en-Josas, France; francois.lefevre@inra.fr

**Keywords:** PRRSV, GP2a, MARC-145, adaptation mutation

## Abstract

The Meat Animal Research Center-145 (MARC-145) cell line has been proven to be valuable for viral attenuation regarding vaccine development and production. Cell-adaptation is necessary for the efficient replication of porcine reproductive and respiratory syndrome virus (PRRSV) in these cells. Multiple sequence analysis revealed consistent amino acid substitutions in GP2a (V88F, M94I, F95L) of MARC-145 cell-adapted strains. To investigate the putative effect of these substitutions, mutations at either position 88, 94, 95, and their combinations were introduced into two PRRSV1 (13V091 and IVI-1173) infectious clones followed by the recovery of viable recombinants. When comparing the replication kinetics in MARC-145 cells, a strongly positive effect on the growth characteristics of the 13V091 strain (+2.1 log10) and the IVI-1173 strain (+1.7 log10) compared to wild-type (WT) virus was only observed upon triple amino acid substitution at positions 88 (V88F), 94 (M94I), and 95 (F95L) of GP2a, suggesting that the triple mutation is a determining factor in PRRSV1 adaptation to MARC-145 cells.

## 1. Introduction

Porcine reproductive and respiratory syndrome virus (PRRSV) is a small enveloped positive-stranded RNA virus assigned to the family, *Arteriviridae*, family, *Nidovirales* [1,2,3]. The PRRSV virion has a diameter ranging from 50 to 70 nm and consists of a phosphorylated nucleocapsid, surrounded by an envelope [4,5,6]. The genome of PRRSV contains multiple functional open reading frames (ORFs). ORF1a and 1b occupy 75% of the genome coding for proteins involved in viral replication and transcription [7,8]. The remaining one-fourth of the viral genome consists of seven relatively small ORFs, encoding the structural proteins. The envelope glycoproteins GP2a and GP4 are membrane proteins encoded by ORF2a and ORF4, respectively [9]. The GP4 protein along with GP2a serves as the viral attachment protein responsible for mediating interactions with the CD163 virus entry mediator [10] and subsequent PRRSV uncoating [11]. GP2a contains an N-terminal signal peptide, an ectodomain, a C-terminal transmembrane segment, and a short cytoplasmic tail. The cysteine residue in the ectodomain of GP2a is supposed to be critical for the formation of intra- and inter-molecular (interaction with GP4 ectodomain) disulphide bridges in EAV [12]. However, it remains unclear if it is similar for PRRSV. GP3, encoded by ORF3, is reported to be anchored late with a preformed GP2a/GP4, forming a heterotrimer complex [13]. However, the exact interactions among the structural proteins remain unclear. A novel structural protein encoded by ORF5a was predicted to be a membrane protein [14]. More recently, nsp2 has been identified as a virion membrane-associated structural PRRSV protein [15].

Like the other members of the *Arteriviridae* family, PRRSV has a restricted cell tropism in vivo. PRRSV is able to replicate primarily in porcine alveolar lung macrophages, differentiated blood monocytes, and monocyte-derived dendritic cells. Macrophages from other sites, like lymph nodes, tonsils, spleen, turbinates, fetal placenta, and choroid plexus, are also susceptible for PRRSV infection [16,17,18,19,20]. Porcine alveolar macrophages (PAM) are known to be permissive to PRRSV infection and may support viral replication. They are currently the most extensively used cells to study relevant aspects of PRRSV replication. Meat Animal Research Center-145 (MARC-145) cells have proven to be valuable for the development and production of vaccines upon adaptation of PRRSV [21,22]. Multiple potential cellular receptors have been identified in the PAM and MARC-145 cells. Among them, heparan sulphate [23], sialoadhesin (Sn, Siglec-1) [24], and Siglec-10 are involved in PRRSV binding to macrophage [25,26]. The GP5/M complex is the viral ligand complex that mediates the initial binding steps [27]. CD163, a member of the scavenger receptor cysteine-rich family, interacts with the viral GP2a/GP4/GP3 complex and is responsible for the disassembly of PRRSV [10]. DC-SIGN, which was found to be expressed in both macrophages and dendritic cells, is reported to be important for the binding and entry of PRRSV. Vimentin (CD151) is found to be important for PRRSV infection on MARC-145 cells [28].

Previously, it was shown that the introduction of a phenylalanine and leucine in the minor glycoprotein, GP2a, at positions 88 and 95, respectively [29], improved the growth of the Lelystad PRRSV1 strain in CL2621 cells. These cells are also derived from the African green monkey cell line (MA-104), like the MARC-145 cells [30]. We observed that PRRSV1 becomes adapted to MARC-145 cells with a higher virus titer after more than 20 serial passages. The aim of this study was to identify the mutations leading to this adaptation.

## 2. Materials and Methods

### 2.1. Cells and Viruses

Primary PAM were isolated from 4- to 6-week-old pigs as described previously [31]. Cells were maintained in Roswell Park Memorial Institute (RPMI) medium containing 10% Fetal Bovine Serum (FBS), 100 U/mL penicillin, and 0.1 mg/mL streptomycin 0.5% gentamycin, 1% tylosin, 1 mM sodium pyruvate and 1% non-essential amino acids. MARC-145 cells were propagated in Eagle’s minimal essential medium supplemented with 10% FBS, 100 U/mL penicillin, and 0.1 mg/mL streptomycin. Virus titers (expressed as tissue culture infectious dose with 50% endpoint [TCID_50_] per mL) were determined in PAM or MARC-145 cells and calculated according to Reed and Muench [32].

Our laboratory isolated and characterized the following PRRSV1 strains: Lena, 08V156, 08VA, and 13V091 [33,34,35]. The accession numbers of these sequences and sequences of other virus strains used in this study (type 1 (*n* = 69) and type 2 (*n* = 638)) are listed in Supplementary Tables S1 and S2.

### 2.2. Serial Passaging of PRRSV Strains and Sequencing

Fixed-volume serial passages were done using a monolayer of MARC-145 cells. After 20–70 passages, sequencing of passaged viruses (08V156 23rd passage, 08VA 52nd passage, Lena 52nd passage, 13V091 73rd passage) was performed. For sequencing of wild-type (WT) and passaged strains, PCR products covering the whole PRRSV genome in two fragments were processed with the use of a 318 v2 chip on the Ion Torrent PGM Benchtop Sequencer (Ion Torrent, Guilford, CT, USA) [36]. We performed an additional quality check of the trimmed reads (FastQC, Babraham bioinformatics, http://www.bioinformatics.babraham.ac.uk). After de novo assembly (CLC Genomics Workbench, CLC bio, http://clcbio.com), the obtained contigs were blasted (blastn, NCBI, http://blast.ncbi.nlm.nih.gov). The sequence with the best percentage of identity to the contigs was used as a reference when ‘mapping reads to reference’. Sequences alignment was done using the Clustal W method built-in the MEGA software [37]. The complete genome sequences of PRRSV1 strains acquired by us and downloaded from GenBank (both at the nucleotide and amino acid levels) were sorted into two groups. The first group contained wild-type field strains. The second group was composed of the strains adapted to MARC cells and the attenuated vaccines. A genome-wide association study (GWA study, or GWAS) was performed based on these two groups. The difference between the field strains and MARC-145 adapted strains was assessed using R script (https://github.com/itrus/GWAS-fasta). The α = 0.01 level for the chi-square test was taken as statistically different in between-group comparisons. Results were illustrated with a Manhattan dot plot.

### 2.3. Site-Directed Mutagenesis in GP2a Using cDNA of Two PRRSV1 Strains

**Figure 1:**
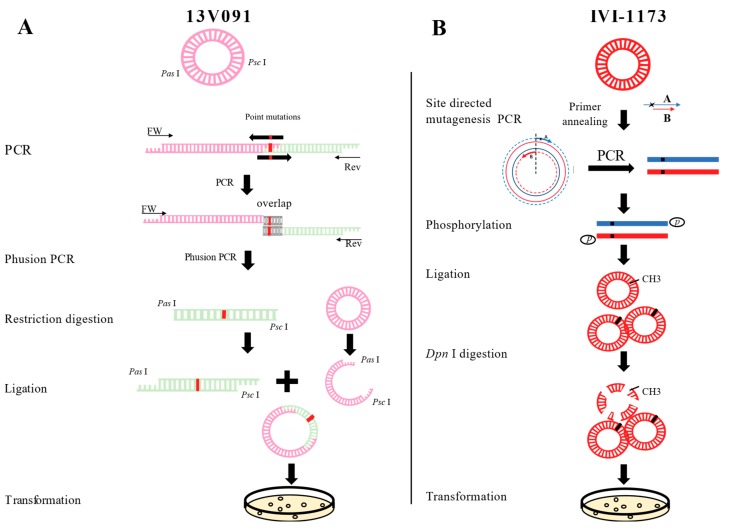
Mutant variants construction strategy for the infectious clone containing the full genome of the 13V091 strain (**A**) and IVI-1173 strains (**B**).

The mutation, V88F, already preexisted in the wild-type 13V091 strain. M94I, F95L in GP2a were introduced individually (Mut94, Mut95) or in combination (Mut9495) into a 13V091 strain plasmidic infectious clone, later designated as FL13 (constructed by F. Lefèvre, unpublished results), bearing the complete cDNA sequence of the 13V091 strain under the control of a eukaryotic promoter. To do this, mutations were first introduced in the cloned *Pas*I-*Psc*I cDNA fragment (containing the GP2a ORF) by fusion PCR-based mutagenesis with the primers listed in Table 1. Phusion High-Fidelity DNA Polymerase (Thermo Fisher Scientific, Waltham, MA, USA) was used for PCR. Mutations were introduced in the cloned wt *Pas*I-*Psc*I fragment by replacing an appropriate restriction fragment by its mutated homologue from the PCR product. Sequences of the three modified *Pas*I-*Psc*I fragments were checked for the presence of only desired mutations. To construct the three mutated infectious clones, the mutated *Pas*I-*Psc*I fragments were then individually introduced into the full-length FL13 infectious clone instead of their wt homologue via *Psc*I-*Pas*I cloning (Figure 1A) using a rapid DNA Ligation kit (Roche, Basel, Switzerland).

The mutations (V88F, M94I, F95L) were individually introduced by the Q5 Site-Directed Mutagenesis Kit (New England Biolab, Ipswich, MA, USA) in the plasmid clone, pIVI-1173 [38] containing the full-length cDNA of the infectious genome of the PRRSV1 IVI-1173 strain [GenBank: KX622783]. Primers were designed through NEBaseChanger (http://nebasechanger.neb.com/) (Table 1). After purification of the PCR product, the template DNA was removed by *Dpn*I (New England Biolab). Before T4 DNA ligation of the PCR product, the fragments were phosphorylated with T4 Polynucleotide Kinase (New England Biolab). Ligation products were transformed into *Escherichia coli* DH10b competent cells (Figure 1). The mutant plasmids designated pFL13-Mut94, pFL13-Mut95, pFL13-Mut9495, pIVI-1173-Mut88, pIVI-1173-Mut94, pIVI-1173-Mut95, pIVI-1173-Mut8894, pIVI-1173-Mut8895, pIVI-1173-Mut9495, and pIVI-1173-Mut889495 were verified by Sanger sequencing.

### 2.4. Generation of Recombinant Viruses

#### 2.4.1. Transfection of DNA Plasmids and Rescue of FL13 Wild-Type and Mutant Constructs

To check if the constructed clones could be successfully expressed, BHK-21 cells were seeded at a concentration of 2 × 10^5^ cells per well in 24-well plates. After 24 h, cells were transfected with 1.6 µg of DNA plasmid mixed with 4 µL Lipofectamine 2000 (Invitrogen, Carlsbad, CA, USA). At 48 h post-transfection, expression of PRRSV N protein was confirmed by immunofluorescence staining using monoclonal antibodies (13E2, IgG2a) against the N protein of PRRSV [39]. The supernatant was collected after one freeze-thaw cycle, clarified by centrifugation, and used to infect PAM. Rescue of infectious virus was confirmed by monitoring N protein expression by IPMA (immunoperoxidase monolayer assay) after 48 h and by virus titration in PAM.

#### 2.4.2. In Vitro Transfection of Transcripts and Rescue of PRRSV1 for IVI-1173 and IVI-1173 Mutant Viruses

The T7 promoter-based infectious clone, pIVI-1173, and the mutant constructs were linearized with the restriction endonuclease, *Swa*I, at the 3′-terminal run-off site (downstream of the polyA tail). Purified linearized DNA served as the template for in vitro transcription of capped RNA using the mMESSAGE mMACHINE Ultra T7 kit (Thermo Fisher Scientific) with m7G (5′) ppp (5′) G cap analog. The reaction mixture was treated with *DNase*I to remove the template DNA and then purified with a RNeasy Mini Kit (Qiagen, Venlo, Netherlands). RNA was resuspended in water and stored at −70 °C. Size, integrity, and concentration of the capped transcripts were determined by electrophoresis and Nanodrop 2000c spectrophotometry (Thermo Fisher Scientific). The virus was rescued by transfection of BHK-21 cells followed by infection of PAM. Briefly, BHK-21 cells were seeded in 2 mL cell culture medium at a concentration of 2 × 10^5^ cells/mL in 6-well plates. After 24 h, cells were transfected with 2 µg of transcripts. PRRSV N protein expression was confirmed by IPMA staining as described previously [40]. At 24 h after transfection and one freeze-thaw cycle, the supernatant was collected, clarified by centrifugation, and used to infect porcine alveolar macrophages (PAM). Rescue of infectious virus was confirmed by monitoring N protein expression by IPMA at 48 h after infection and by virus titration in PAM.

### 2.5. Testing Mutant Variants In Vitro in PAM and MARC-145 Cells

Replication kinetics of recombinant viruses was assessed in order to determine the effect of the introduced mutations on the viral replication in vitro. Specifically, the supernatant from BHK-21 cells transfected with either the mutated or the wild-type infectious cDNA clones was passaged on PAM to increase virus yield. Both PAM and MARC-145 cell cultures were inoculated with either wild-type virus (recovered from the original infectious cDNA clone) of IVI-1173 and 13V091 (from plasmid FL13), mutant variants of IVI-1173 (Mut88, Mut95, Mut8894, Mut889495), and 13V091 (Mut94, Mut95, Mut9495), and the original virus stock of 13V091 at a multiplicity of infection (MOI) of 0.1. Cells were incubated for 1 h on a rocking platform (10 rpm). The inoculum was removed, cells were flushed two times with PBS, and 4 mL of fresh cell culture medium was added. Supernatants were collected on a daily base and titrated both on MARC-145 and PAM cells.

### 2.6. Virus Internalization and Entry Assay

PRRSV completes its life cycle within 12 h [41], indicating that infection within this time period is caused by the primary virus penetration and not by newly produced virions or cell-associated spread of the virus. A one-step growth cycle was performed with the recombinant viruses to analyze the differences in virus entry in MARC-145. In parallel, the internalized virus particles per cell were counted. Briefly, MARC-145 cells were infected with wild-type and mutant variants at a MOI of 0.1. After 1 and 12 h post-infection (hpi), cells were fixed with ice cold (−20 °C) methanol prior to immunofluorescence staining with primary monoclonal antibodies 13E2 against PRRSV nucleocapsid protein (IgG2a, 1:50) [38]. As control, 1C11 against gB of PrV (IgG2a, 1:50) was used as isotype matched irrelevant antibody [42]. As a secondary antibody, fluorescein isothiocyanate (FITC)-conjugated polyclonal goat anti-mouse IgG antibodies (1:400) (Invitrogen) were used. For the internalization assay, cell cortical actin was stained with Texas Red™-X Phalloidin (Thermo Fisher Scientific) to differentiate bound and internalized particles. Cell nuclei were stained with Hoechst 33342. The percentage of PRRSV infected cells was quantified with a fluorescence microscope (Leica Microsystems GmbH, Wetzlar, Germany). The number of virus particles present inside the cortical actin ring was counted; 15 cells were selected for counting in each experimental condition. The percentage of positive cells was calculated in 20 randomly selected fields, covering an area of 0.55 mm^2^ per field.

### 2.7. Statistical Analysis

The virus entry assay for the recombinant PRRSV variants was performed with at least three replicates. The difference in the infection rate and number of internalized virus particles between the variants and WT was compared using a one-way analysis of variance (ANOVA) followed by Sidak’s multiple comparisons test. Differences were considered statistically significant at *p* < 0.05.

## 3. Results

### 3.1. Identification of Several Adaptation-Related Amino Acid Mutations in the PRRSV1 Glycoprotein

**Figure 2:**
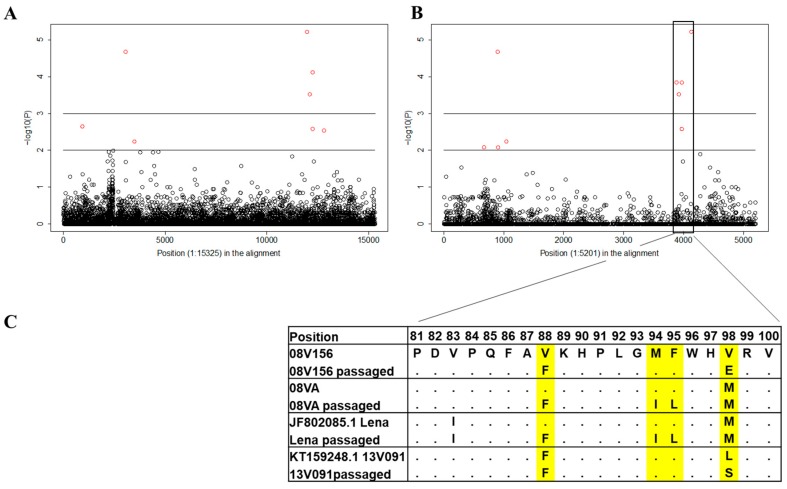
Manhattan plot with comparison results for Porcine reproductive and respiratory syndrome virus 1 (PRRSV1) full genome sequences on nucleotide (**A**) and amino acid (**B**) levels. Horizontal lines are plotted at *p* = 0.01 and *p* = 0.001 levels. (**C**) The fragment of the GP2a protein containing different amino acid residues in the wild-type and MARC-145 passaged strains.

The GWA study compared eight MARC-145-adapted virus sequences with the sequences of 70 field viruses. Two single-nucleotide polymorphisms (SNPs) with significantly altered allele frequency between the two groups were identified. These SNPs were located in the gene encoding Nsp2 and GP2a. (Figure 2A,B). Further analysis revealed that three amino acid mutations (V88F, M94I, and F95L) in GP2a were acquired by the passaged strains at the identical position as in attenuated subtype 1 PRRSV1 strains (MLV-DV [GenBank: KF991509.2] and MLV-VP046 [GenBank: KJ127878.1]). Verheije et al. previously showed that the V88F and F95L mutations induced improved viral growth in CL2621 cells (MA-104 derivative cell line) [29]. In this study, sequence alignment of multiple wild-type and MARC-145 cell-adapted PRRSV1 strains revealed an additional recurrent amino acid substitution located in GP2a at amino acid position 94 (Figure 2C). Thus, several consistent mutations identified with different virus strains upon serial passaging in the MARC-145 cell line suggest that they play a role in the adaptation to this cell line.

### 3.2. Construction and Recovery of 13V091 and IVI-1173 WT and their Corresponding Mutant Variants

To investigate the putative effect of the substitutions at these positions, the aforementioned mutations were introduced in the background of a full-length infectious cDNA clone of the PRRSV1 13V091 and IVI-1173 strains via site-directed mutagenesis. The amino acid substitution at position 88 (V88F) was present in the GP2a region of the infectious cDNA clone of the 13V091 strain ab initio. Introduction of a single amino acid substitution at either position 94 (M94I) or 95 (F95L) and a double substitution was followed with the recovery of viable recombinant viruses denoted as Mut94, Mut95, and Mut9495, respectively. Since there was no substitution at position 88 for the IVI-1173 strain, introduction of a single amino acid substitution at either position 88 (V88F), 94 (M94I), or 95 (F95L), three double substitutions (V88F + M94I, V88F + F95L, and M94I + F95L), and one triple substitution (V88F + M94I + F95L) was performed by site directed mutagenesis of the infectious clone to generate recombinant viruses denoted as Mut88, Mut94, Mut95, Mut8894, Mut8895, Mut9495, and Mut889495, respectively. Subsequent sequencing of the mutated plasmids confirmed the presence of the introduced mutations. To check if the constructed clones were functional, BHK-21 cells were transfected with plasmid DNA (FL13-derived clones) or with in vitro transcribed RNA (pIVI-1173-derive clones) of the mutated infectious clones and with the wild-type virus as the positive control. At 48 h post-transfection, IFA (immunofluorescence assay) was performed to examine the viral protein expression. For 13V091 (FL13-derived), all the mutant variants, as well as the WT, showed evident signal for viral protein staining at 48 hpi. For IVI-1173, the mutant constructs showed a positive signal for viral protein staining except for Mut94 and Mut9495 (Table 2). To check the infectivity of the recovered viruses, the supernatants collected from the transfected BHK-21 cells were used to infect PAM and MARC-145 cells. As shown in Table 2, mutants generated from the 13V091 strain, except Mut94, were able to infect both PAM and MARC-145 cells. Similarly, for the IVI-1173 strain, Mut94 and Mut9495 were negative in BHK-21 transfected cells and consequently also after inoculation of MARC-145 and PAM cell cultures. Mut8895 showed viral protein expression in transfected BHK-21 cells, but was not infectious in MARC-145 and PAM cells. The viable mutant constructs (the 13V091 strain: Mut94, Mut95, and Mut9495; the IVI-1173 strain: Mut88, Mut95, Mut8894, Mut889495) were further passaged on PAMs to increase the virus yield. Viral RNA was extracted and used to confirm the presence of the target mutations in the recovered virus. The different viable mutants (for the 13V091 strain: Mut94, Mut95, and Mut9495; for the IVI-1173 strain: Mut88, Mut95, Mut8894, Mut889495) were further characterized in the following experiments.

### 3.3. Mutations V88F, M94I, and F95L in GP2a do not Affect the Growth Phenotype of Type 1 PRRSVs in PAM

**Figure 3:**
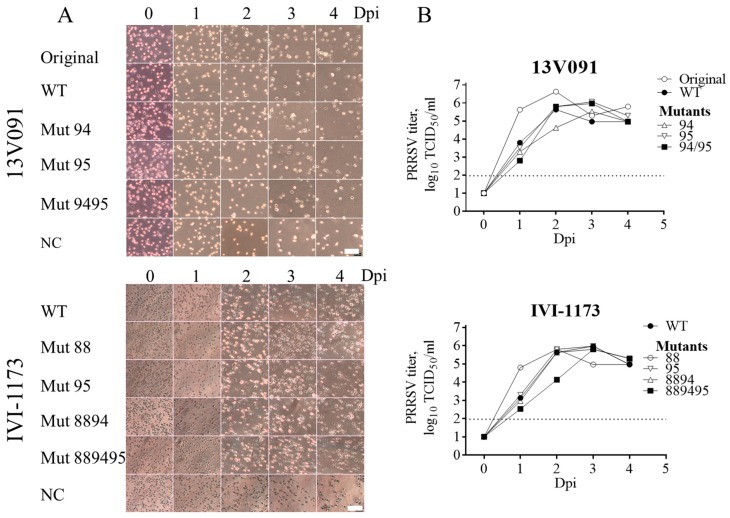
(**A**) Cytopathic effects (CPE) in PAM cells at 0–4 days post inoculation with the original 13V091 or rescued virus from the infectious clones (WT) and mutants of strains 13V091 and IVI-1173. Scale bar = 100 μm (**B**) Virus replication kinetics in PAM. The dotted line represents the limit of detection for the assay.

To determine whether the mutations in GP2a had any effect on the primary cells, PAM were inoculated with the mutant constructs, as well as the wild-type viruses at a MOI of 0.1. The replication kinetics were assessed on PAM. CPE (cytopathic effect) was checked on a daily basis. Virus production was measured from 0 to 4 days post-infection with titration of supernatants on PAMs. Based on the results shown in Figure 3, no differences could be observed when comparing CPE for both 13V091 and IVI-1173 (Figure 3). All the virus variants caused cell death starting from 1–2 days post-infection. The multi-step growth curves of wild-type and mutants were similar. The peak titers for 13V091 WT (5.6 log_10_ TCID_50_/mL) and its mutant variants were similar (Mut94 − 5.5 log_10_ TCID_50_/mL, Mut95 − 6.1 log_10_ TCID_50_/mL, and Mut9495 − 6.0 log_10_ TCID_50_/mL). The original 13V091 replicated somewhat better than the rescued variants (Figure 3B). The peak titers on PAM for IVI-1173 were similar as well (WT − 6.0 log_10_ TCID_50_/mL, Mut88 − 5.8 log_10_ TCID_50_/mL, Mut95 − 6.0 log_10_ TCID_50_/mL, Mut8894 − 5.8 log_10_ TCID_50_/mL, Mut889495 − 5.8 log_10_ TCID_50_/mL).

### 3.4. Mutations V88F, M94I, and F95L in GP2a are Important for Adaptation of Type 1 PRRSV to MARC-145 Cells

**Figure 4:**
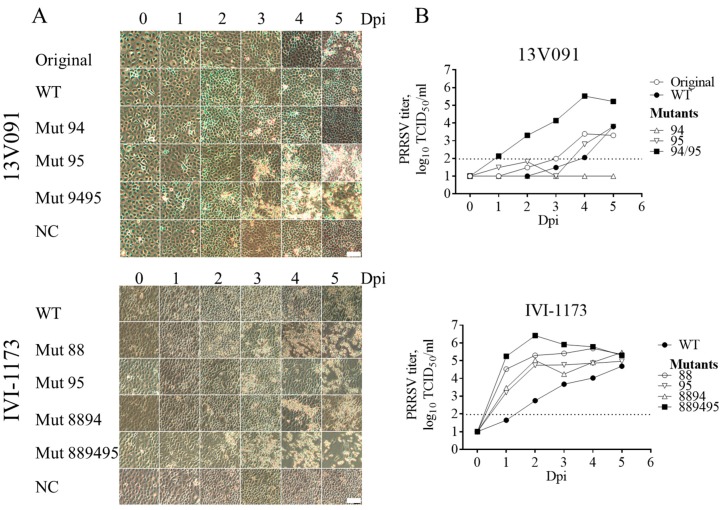
(**A**) CPE in MARC-145 cells at 0–5 days post inoculation with the original 13V091 or rescued virus from the infectious clones (WT) and mutants of strains 13V091 and IVI-1173. Scale bar = 100 μm (**B**) Virus replication kinetics in MARC-145 cells. The dotted line represents the limit of detection for the assay.

To assess whether mutations in GP2a will have an effect on the replication of the virus in MARC-145 cells, these cells were inoculated with original and rescued strains at a MOI of 0.1. The presence of CPE was controlled daily (Figure 4). For the 13V091 strain, the WT, as well as Mut94 (variant with V88F, M94I mutations) cDNA derived variant showed no obvious CPE at 0–5 days post-infection (dpi). In contrast, Mut95 (variant with V88F, F95L mutations) showed a clear CPE starting from 3 days post-infection. Mut9495 (variant with V88F, M94I, and F95L mutations) showed the earliest CPE (2 dpi). For the IVI-1173 strain, the WT showed CPE at 5 dpi. Mut88 and Mut95 have already shown clear CPE at 3 days post infection. Mut8894 showed CPE at 4 dpi, while Mut889495 showed the earliest CPE at 2 dpi (Figure 4). Comparison of the replication kinetics in MARC-145 cells (Figure 4) revealed a positive effect (+0.8 log_10_ compared to the wild-type virus) of the growth characteristics upon introduction of a single amino acid substitution at position 95 of the GP2a in the 13V091 strain genome. On the contrary, a negative effect could be seen when introducing a mutation at position 94 (≤−2.0 log_10_). Interestingly, the double mutant, Mut9495, showed the highest viral replication compared to the other virus variants (+2.2 log_10_). Moreover, for IVI-1173, positive effects were observed upon introduction of a single mutation at position 88 (+1.2 log_10_ compared to the wild-type virus) and 95 (+0.3 log_10_) as well as double substitutions at positions 88 and 94 (+0.5 log_10_) of the GP2a. Similarly, triple substitutions at positions 88, 94, and 95 (Mut889495) of GP2a showed the highest (+1.7 log_10_) and also the earliest viral replication compared to the other viruses, suggesting that the triple mutation at these positions is the most optimal adaptation of PRRSV1 to the MARC-145 cell line. Viral titers were in agreement with the observed CPE in MARC-145 cell line (Figure 4).

### 3.5. Mutations V88F, M94I, and F95L in GP2a Improves the Entry of PRRSV1 in MARC-145 Cells

**Figure 5:**
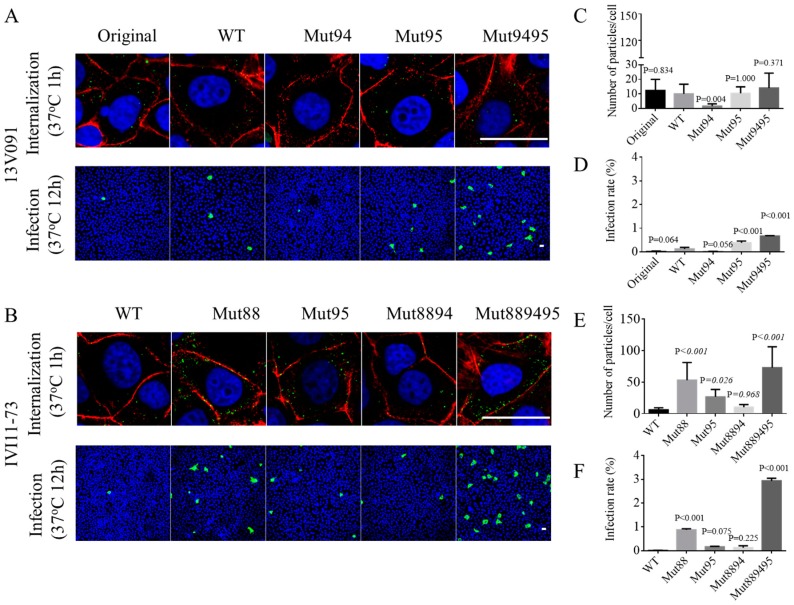
Immunofluorescence staining of internalized PRRSV particles 1hpi and infected MARC-145 cells at 12 hpi. Cells were inoculated with 13V091 (**A**) or IVI-1173 (**B**) original strain, WT, or their mutant variants and fixed at 1 and 12 hpi. Immunofluorescence assay was performed with mAb 13E2 against PRRSV nucleocapsid protein. Scale bar: 30 μm. 15 cells were selected for counting the internalized particles for each condition (**C**,**E**). The percentage of infected cells was counted in 20 randomly selected fields covering an area of 0.55 mm^2^ (**D**,**F**). Data were represented as M ± SD for three repeats. *p* values were shown for each recombinant variant or original virus in comparison with WT.

In order to analyze the role of mutations in GP2a for the primary steps of viral replication, internalized virus particles per cell were quantified at 1 hpi and infection kinetics was assessed at 12 hpi. This timepoints within one replication cycle reflects the efficiency of primary replication steps of the virus, including cell entry. MARC-145 cells were infected with all generated virus variants. Then, at 1 and 12 hpi, cells were fixed for staining of PRRSV nucleocapsid protein. As it is shown in Figure 5, a single amino acid substitution at position 95 in GP2a of the 13V091 strain significantly improved the infectivity rate compared to the WT (WT: 0.13 ± 0.07%, Mut95: 0.38 ± 0.07%, *p* = 0.0002, ANOVA). A single amino acid substitution at position 94 provided the lowest infectivity rate of 0.01 ± 0.01%. The double substitution at positions 94 and 95 (Mut9495) showed the highest infection rate compared to the other virus variants (0.67 ± 0.02%, *p* < 0.0001). For the internalization assay, though no significant difference was observed, the Mut9495 showed the highest number of internalized particles compared to the rest groups. For the IVI-1173 strain, the introduction of a single mutation at position 88 significantly improved the infectivity rates (Mut88: 0.88 ± 0.04%, WT: 0.01 ± 0.01%, *p* < 0.0001). No significant differences were observed for a single substitution at position 95 (Mut95: 0.16 ± 0.03%, WT: 0.01 ± 0.01%, *p* = 0.075) and double substitutions at position 88 and 94 (Mut8894: 0.12 ± 0.09% WT: 0.01 ± 0.01%, *p* = 0.225). Similar to the 13V091, the triple substitution at position 88, 94, and 95 (Mut889495) provided the highest infection rate (Mut889495: 2.94 ± 0.11%, WT: 0.01 ± 0.01%, *p* < 0.0001). For the internalization assay of IVI-1173: Mut88, Mut95, and Mut889495 showed a significantly higher number of internalized virus particles (Mut88: 53 ± 27 particles/cell, *p* < 0.0001; Mut95:27 ± 11 particles/cell, *p* = 0.026; and Mut889495:73 ± 32 particles/cell, *p* < 0.0001) compared to WT (6 ± 3 particles/cell).

## 4. Discussion

Viruses, in particular, RNA viruses, possess relatively high mutational rates [43,44]. Although the speed of mutations and evolution can change depending on many factors, viruses overall have higher chances for mutations when the host environment is changing. This elevated mutation rate allows viruses to quickly adapt to changing conditions in their new host cell. Mutations conferring cell culture adaptation with enhanced virus replication have been described for several viruses, such as Hepatitis C virus, Infectious bursal disease virus and Sindbis virus [45,46,47]. Recently, Zhang et al. demonstrated that minor envelope proteins, GP2a-GP3, are important for the adaptation of type 2 PRRSV strains in MARC-145 cells [48]. Moreover, Verheije et al. [29] showed that two amino acid mutations (V88F and F95L in GP2a) improved viral growth for the type 1 Lelystad strain in CL2621 cells (MA-104 derivative cell line). In the present study, we serially passaged several type 1 PRRSV strains on MARC-145. Then, based on the genome sequences’ comparison between the cell adapted strains and wild-type field isolates, an additional mutation site (M94I in GP2a) was found. In combination with V88F and F95L, it was observed in two out of four studied MARC-145 adapted strains: Lena and 08VA (Figure 2). The triple mutations (V88F, M94I, and F95L) in GP2a were also observed in ESP-1991-Olot91, which is a MARC-145 adapted version of the Olot91 isolate [49], the MARC-145 adapted modified live-virus vaccine strains, Amervac PRRS, and two type 1 PRRSV strains (SD01-08 and GZ11-G1) isolated in the US and China, respectively, and adapted to MARC-145 [50] (Supplementary Table S3). In conclusion, the data presented above demonstrated that V88F, M94I, and F95L are likely associated with the MARC-145 cell line adaptation for type 1 PRRSV strains.

**Figure 6:**
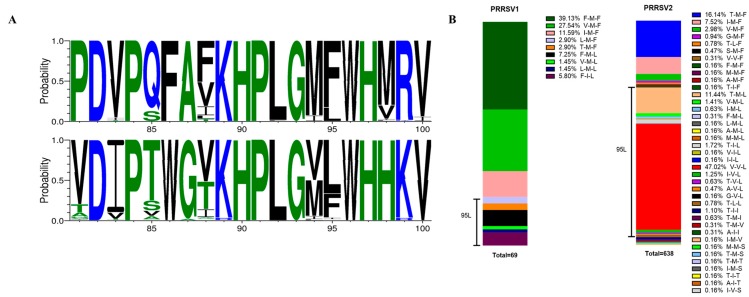
A graphical representation of an amino acid multiple sequence alignment of the GP2a protein (fragment covering amino acid residues at positions 81–100) from type 1 (*n* = 69) and type 2 (*n* = 638) PRRSVs (**A**). Logo image consists of stacks of symbols, one stack for each position in the sequence. The height of symbols within the stack indicates the relative frequency of each amino acid at that position [51]. (**B**) The percentage of different GP2a phenotypes were presented with bar charts for PRRSV1 and PRRSV2.

Cloned infectious full-length genomes are valuable tools for studying various aspects of numerous RNA viruses, including PRRSV. They allow manipulations of the viral genome and direct production of recombinant infectious virus progeny. To confirm the aforementioned hypothesis, two type 1 PRRSV infectious clones based on the 13V091 and IVI-1173 strains were used in this study. Mutations V88F, M94I, and F95L were introduced in GP2a individually or in combination in the 13V091 and IVI-1173 infectious clones. Upon transfection of BHK cells with cDNA or mRNA, except IVI-1173-Mut94 and IVI-1173-Mut9495, all of the other mutant recombinant and wild-type variants were successfully expressed and recovered. Transferring recovered viruses to PAM and MARC-145 cells resulted in an impaired replication of 13V091-Mut94 (Mut8894) in MARC-145 cells (Figure 4). The presence of phenylalanine at position 88, isoleucine at position 94, and leucine at position 95 in field virus sequences differed. PRRSV field sequences showed a high frequency (36/69) of phenylalanine at position 88 in type 1 PRRSVs, but not in type 2 PRRSVs (3/638) (Figure 6). Leucine at position 95 could be found in type 2 PRRSV field sequences at a high prevalence (425/638), but not in type 1 PRRSV field sequences (12/69). Presence of isoleucine at position 94 of GP2a is rare (type 1–5/69; type 2–25/638). Notably, isoleucine at position 94 in PRRSV1 field sequences is always combined (5/5) with leucine at position 95. Thus, although it seems not a frequent situation in field PRRSV1 sequences to find isoleucine at the position 94, it is always combined with leucine at position 95. A combined effect of these two mutations could be considered. The IVI1173-Mut94 (V-I-F) GP2a phenotype that could not be rescued in this study cannot be found in both analyzed type 1 (0/69) and type 2 (0/638) PRRSV field strains (Figure 6 and Supplementary Table S4), indicating the unfavorable phenotype for both genotypes. Because the majority of the type 2 strains have a V-V-L phenotype (300/638) and X-M-F phenotype (182/638) and not a F-I-L phenotype, we may not easily explain why most type 2 strains are ready to replicate in MARC-145 cells without adaptation. Nevertheless, we observe that one of the three important residues for type I adaptation, namely leucine at position 95, showed a much higher percentage in type 2 compared to type 1 PRRSV strains (Figure 6B and Supplementary Table S4).

**Figure 7:**
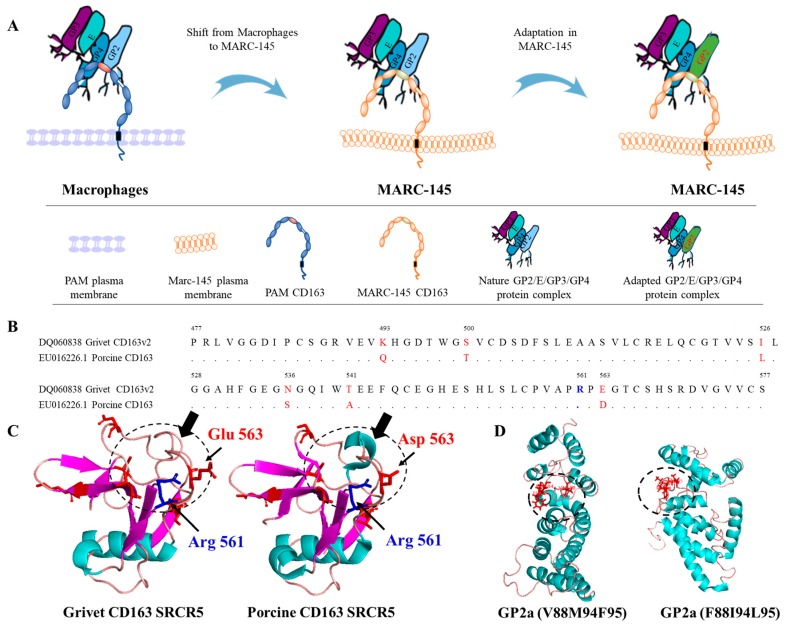
Schematic representation of the possible mechanism of PRRSV1 adaptation to MARC-145 (**A**). Virus, originally replicating in macrophages, was shifted to MARC-145 cells. An infection will be completed only with an appropriate cellular entry mediator. CD163 is the essential cellular receptor that is present in both cell types [54]. Its SRCR5 domain interacts with the viral minor glycoprotein complex (GP2a/E/GP3/GP4) during the infection [10,11]. An amino acid sequence comparison of the SRCR5 domain of the porcine CD163 and simian CD163 is shown in (**B**). (**C**) The structure of porcine CD163 SRCR5 (5jfb) was used as a template for building the MARC-145 CD163 SRCR5 domain with SWISS-MODEL. Red labeled residues indicated the residues which differ between the porcine and grivet. Structural differences were indicated with black arrows. The important PRRSV binding site (Arg561) and differences close to the binding site: Glu563 in grivet CD163 SRCR5 and Asp563 in porcine CD163 SRCR5 were highlighted. When the host environment changes (from porcine CD163 to grivet CD163), it will result in amino acid mutations of its interacting ligand viral protein (e.g., GP2a) according to the results described in the present study. (**D**) The structure of GP2a with V-M-F phenotype (left) and F-I-L phenotype (right) were predicted with I-TASSER (https://zhanglab.ccmb.med.umich.edu/I-TASSER/). The mutated residues were indicated in red.

Comparison of the replication kinetics in MARC-145 cells (Figure 4) demonstrated the highest viral replication for both the 13V091 strain (+2.2 log10) and the IVI-1173 strain (+1.7 log10) when triple substitutions were present (V88F, M94I, and F95L). This suggests that the simultaneous introduction of three mutations at positions 88, 94, and 95 is a determining factor in PRRSV1 adaptation to the MARC-145 cell line. The replication process for PRRSV in PAM has been clearly demonstrated before [52]. Initially, the PRRSV virion attaches to the macrophage surface via heparan sulphate. Subsequently, the virus binds to the Siglec receptor via M/GP5 glycoprotein complexes [27]. Upon attachment to the Siglec receptor, the virus-receptor complex is internalized and this step is followed by viral genome release, thereby initiating the transcriptional steps. The scavenger receptor, CD163, is essential for the PRRSV fusion and genome release [53] and exert its function through interaction with GP2a and GP4 [10]. The low expression level of CD163 in MARC-145 cells is sufficient to render them susceptible to PRRSV infection [54]. CD163 from MARC-145 cells share 87.6% amino acid identity with CD163 from PAM. The SRCR5 domain is the most important domain that is involved in the interaction with the viral minor glycoproteins complex [11]. Several variations (6/101) were observed in this domain (Q493K, T500S, L526I, S526N, A541T, and D563E) (Figure 7) between pig and grivet. The crystal structure of the SRCR5 domain of porcine CD163 has been determined recently, and the arginine residue at position 561 (Arg561) has been demonstrated to be important for PRRSV infection [55]. Based on this structure, we built the predicted structure of grivet CD163 SRCR5 domain using the SWISS-MODEL (https://swissmodel.expasy.org/; Figure 7C). One major change is the mutation of Asp into Glu at position 563, in close vicinity to Arg561. Both Asp and Glu are negatively charged residues, but Glu has a longer side chain, therefore, the localization of the negative charge will be changed. This change next to a residue important for the binding of PRRSV (Arg561) would force the virus to adapt. Further analysis of the modeled structure of the grivet CD163 SRCR5 domain predicts the loss of one beta-sheet close to Arg561 compared to porcine CD163. Based only on this model and because the structure of the viral ligand complex is unknown, it would be too speculative to discuss about the precise role of this change and the other mutations in the observed adaptation of GP2a. Based on the GWAS study, only GP2a, but not Gp4, has shown significant residue variation between the MARC-145 adapted strains compared to the wild type strains, indicating that GP2a plays a preponderant role in the interaction with CD163. The triple substitution in GP2a allowed mutant recombinant viruses to show the highest infection rate as well as the highest number of internalized particles for both the 13V091 and IVI-1173 strains (Figure 5). This result suggests that the triple substitution might allow that GP2a conformational changes that favors the interaction with the MARC-145 CD163 SRCR5 binding domain. In the absence of structural data for GP2a, we discuss below the putative impact of the mutations. The substitutions (V88F, M94I, and F95L) are all changes within the hydrophobic and neutral amino acids group, which will not cause charge changes of the protein. Introduction of the triple mutation in the predicted (I-TASSER) structure of wild-type GP2a resulted in a quite different structure (Figure 7D). V88F may compensate for the loss of a phenylalanine at position 95 (F95L). Because of the presence of a lysine at position 89, a stabilizing cation-π interaction between F88 and K89 is possible [56]. Based on the analysis, these differences in the SRCR5 binding domain of CD163 between PAM and MARC-145 might be the driving force for the adaptation changes of the GP2a ligand (Figure 7).

Currently, we can only speculate about the molecular mechanism of cell culture adaptation. One of the hallmarks of PRRSV is its remarkable genetic diversity due to the low fidelity and a lack of proofreading activity by its RNA-dependent RNA polymerase [UniProt: B2BLJ4] [57]. A quasispecies theory explaining genetically variable RNA viruses may be a possible explanation here [58]. According to this theory, a viral quasispecies could be defined as a group of genetically related viruses that are closely distributed around a consensus sequence and, for any given environment, collectively contribute to the characteristics of the whole group [58,59]. The existence of PRRSV strains as a quasispecies population allows rapid adaptation to changing conditions without ad hoc mutagenesis. Individually infected pigs could harbor multiple intra-strain variants at the same time [60]. Although next-generation sequencing techniques allow identification of multiple single-nucleotide variants in the PRRSV genome [49], the impact of these variants on interactions of the host cell and viral proteins via molecular modeling should be established to understand the link between the micro-evolutionary events of PRRSV strains.

## 5. Conclusions

In this study, we observed that adaptation of PRRSV1 strains to MARC-145 cells was combined with several single nucleotide mutations in the viral genome based on GWAS. Three nucleotide mutations in GP2a, leading to three amino acid substitutions (V88F, M94I, and F95L in GP2a), were observed. In vitro site-directed mutagenesis and reverse genetics analyses showed that the adaptation mechanism of PRRSV1 strains is based on these molecular changes. The results obtained within this study allow in silico cell adaptation of PRRSV1 strains and efficient production of virus, providing a more efficient way for the development and production of PRRSV vaccines.

## Figures and Tables

**Table 1:** Primers used for the mutagenesis of FL13 and IVI-1173 infectious clones.

Name of the Primer	Sequence (5′-3′)
FL13 AVRup_D	GACGTCAGAGCACCACGGTTGGAGGG
FL13_94I_D	AAGCACCCCTTGGGTATaTTTTGGCACTTGCGGGTTTCC
FL13_94I_R	GGAAACCCGCAAGTGCCAAAATATACCCAAGGGGTGCTT
FL13_95I_D	AAGCACCCCTTGGGTATGcTTTGGCACTTGCGGGTTTCC
FL13_95I_R	GGAAACCCGCAAGTGCCAAAGCATACCCAAGGGGTGCTT
FL13_9495I_D	AAGCACCCCTTGGGTATacTTTTGGCACTTGCGGGTTTCC
FL13_9495I_R	GGAAACCCGCAAGTGCCAAAAGTATACCCAAGGGGTGCTT
FL13_F3Psc_R	GGAATGGTCATAGACAGTTCCGCCGGCGCGCGCGCCTCACACGTAGAGCTTCC
pIVI-1173 Mut88-F	ACAATTTGCGtTTAAGCACCC
pIVI-1173 Mut88-R	GGAACATCCGGTCTACAG
pIVI-1173 Mut94-F	CATTGGGCATaTTTTGGCACATG
pIVI-1173 Mut94-R	GGTGCTTAACCGCAAATTG
pIVI-1173 Mut95-F	ATTGGGCATGcTTTGGCACAT
pIVI-1173 Mut95-R	GGGTGCTTAACCGCAAATT
pIVI-1173 Mut88 94-F	CATTGGGCATaTTTTGGCACATG
pIVI-1173 Mut88 94-R	GGTGCTTAAACGCAAATTG
pIVI-1173 Mut88 95-F	ATTGGGCATGcTTTGGCACAT
pIVI-1173 Mut88 95-R	GGGTGCTTAAACGCAAATTG
pIVI-1173 Mut88 9495-F	CATTGGGCATacTTTGGCACATG
pIVI-1173 Mut88 9495-R	GGTGCTTAAACGCAAATTG

**Table 2:** Viral protein expression and infectivity of the mutant constructs.

13V091					IVI-1173				
Constructs	GP2a Phenotype ^1^	BHK Cells ^2^	Infectivity in PAM	Infectivity in MARC-145	Constructs	GP2a Phenotype	BHK Cells	Infectivity in PAM	Infectivity in MARC-145
					WT	V-M-F	+	+	+
WT	F-M-F	+	+	+	Mut 88	F-M-F	+	+	+
					Mut 94	V-I-F	-	-	-
					Mut 95	V-M-L	+	+	+
Mut 94	F-I-F	+	+	±	Mut 8894	F-I-F	+	+	+
Mut 95	F-M-L	+	+	+	Mut 8895	F-M-L	+	-	-
					Mut 9495	V-I-L	-	-	-
Mut 9495	F-I-L	+	+	+	Mut 889495	F-I-L	+	+	+

^1^ Amino acid residues at positions 88, 94, and 95 of GP2a; ^2^ PRRSV viral protein expression.

## Supplementary Materials

The following are available online at http://www.mdpi.com/1999-4915/11/1/36/s1, Supplementary Table S1. Information and accession numbers of type 1 PRRSV GP2a; Supplementary Table S2. Information and accession numbers of type 2 PRRSV GP2a; Supplementary Table S3. Amino acid sequences of the GP2a fragment of the MARC-145 adapted strains; Supplementary Table S4. Summary of the GP2a phenotype for type 1(*n* = 69) and type 2 (*n* = 638) PRRSV strains available in GenBank

## References

[1] Conzelmann K.K., Visser N., Van Woensel P., Thiel H.J. (1993). Molecular characterization of porcine reproductive and respiratory syndrome virus, a member of the arterivirus group. Virology.

[2] Cavanagh D. (1997). Nidovirales: A new order comprising Coronaviridae and Arteriviridae. Arch. Virol..

[3] Snijder E.J., Meulenberg J.J. (1998). The molecular biology of arteriviruses. J. Gen. Virol..

[4] Benfield D.A., Nelson E., Collins J.E., Harris L., Goyal S.M., Robison D., Christianson W.T., Morrison R.B., Gorcyca D., Chladek D. (1992). Characterization of swine infertility and respiratory syndrome (SIRS) virus (isolate ATCC VR-2332). J. Vet. Diagn. Investig..

[5] Meulenberg J.J., Hulst M.M., de Meijer E.J., Moonen P.L., den Besten A., de Kluyver E.P., Wensvoort G., Moormann R.J. (1993). Lelystad virus, the causative agent of porcine epidemic abortion and respiratory syndrome (PEARS), is related to LDV and EAV. Virology.

[6] Spilman M.S., Welbon C., Nelson E., Dokland T. (2009). Cryo-electron tomography of porcine reproductive and respiratory syndrome virus: Organization of the nucleocapsid. J. Gen. Virol..

[7] Meulenberg J.J. (2000). PRRSV, the virus. Vet. Res..

[8] Fang Y., Snijder E.J. (2010). The PRRSV replicase: Exploring the multifunctionality of an intriguing set of nonstructural proteins. Virus Res..

[9] Dokland T. (2010). The structural biology of PRRSV. Virus Res..

[10] Das P.B., Dinh P.X., Ansari I.H., de Lima M., Osorio F.A., Pattnaik A.K. (2010). The minor envelope glycoproteins GP2a and GP4 of porcine reproductive and respiratory syndrome virus interact with the receptor CD163. J. Virol..

[11] Van Gorp H., Van Breedam W., Van Doorsselaere J., Delputte P.L., Nauwynck H.J. (2010). Identification of the CD163 protein domains involved in infection of the porcine reproductive and respiratory syndrome virus. J. Virol..

[12] Wieringa R., de Vries A.A., Rottier P.J. (2003). Formation of disulfide-linked complexes between the three minor envelope glycoproteins (GP2b, GP3, and GP4) of equine arteritis virus. J. Virol..

[13] Veit M., Matczuk A.K., Sinhadri B.C., Krause E., Thaa B. (2014). Membrane proteins of arterivirus particles: Structure, topology, processing and function. Virus Res..

[14] Oh J., Lee C. (2012). Proteomic characterization of a novel structural protein ORF5a of porcine reproductive and respiratory syndrome virus. Virus Res..

[15] Kappes M.A., Miller C.L., Faaberg K.S. (2013). Highly divergent strains of porcine reproductive and respiratory syndrome virus incorporate multiple isoforms of nonstructural protein 2 into virions. J. Virol..

[16] Duan X., Nauwynck H.J., Pensaert M.B. (1997). Virus quantification and identification of cellular targets in the lungs and lymphoid tissues of pigs at different time intervals after inoculation with porcine reproductive and respiratory syndrome virus (PRRSV). Vet. Microbiol..

[17] Duan X., Nauwynck H.J., Pensaert M.B. (1997). Effects of origin and state of differentiation and activation of monocytes/macrophages on their susceptibility to porcine reproductive and respiratory syndrome virus (PRRSV). Arch. Virol..

[18] Wang X., Eaton M., Mayer M., Li H., He D., Nelson E., Christopher-Hennings J. (2007). Porcine reproductive and respiratory syndrome virus productively infects monocyte-derived dendritic cells and compromises their antigen-presenting ability. Arch. Virol..

[19] Chang H.C., Peng Y.T., Chang H.L., Chaung H.C., Chung W.B. (2008). Phenotypic and functional modulation of bone marrow-derived dendritic cells by porcine reproductive and respiratory syndrome virus. Vet. Microbiol..

[20] Karniychuk U.U., Saha D., Geldhof M., Vanhee M., Cornillie P., Van den Broeck W., Nauwynck H.J. (2011). Porcine reproductive and respiratory syndrome virus (PRRSV) causes apoptosis during its replication in fetal implantation sites. Microb. Pathog..

[21] An T.Q., Tian Z.J., Zhou Y.J., Xiao Y., Peng J.M., Chen J., Jiang Y.F., Hao X.F., Tong G.Z. (2011). Comparative genomic analysis of five pairs of virulent parental/attenuated vaccine strains of PRRSV. Vet. Microbiol..

[22] Tian D., Wei Z., Zevenhoven-Dobbe J.C., Liu R., Tong G., Snijder E.J., Yuan S. (2012). Arterivirus minor envelope proteins are a major determinant of viral tropism in cell culture. J. Virol..

[23] Delputte P.L., Vanderheijden N., Nauwynck H.J., Pensaert M.B. (2002). Involvement of the matrix protein in attachment of porcine reproductive and respiratory syndrome virus to a heparinlike receptor on porcine alveolar macrophages. J. Virol..

[24] Vanderheijden N., Delputte P.L., Favoreel H.W., Vandekerckhove J., Van Damme J., van Woensel P.A., Nauwynck H.J. (2003). Involvement of sialoadhesin in entry of porcine reproductive and respiratory syndrome virus into porcine alveolar macrophages. J. Virol..

[25] Xie J., Christiaens I., Yang B., Breedam W.V., Cui T., Nauwynck H.J. (2017). Molecular cloning of porcine Siglec-3, Siglec-5 and Siglec-10, and identification of Siglec-10 as an alternative receptor for porcine reproductive and respiratory syndrome virus (PRRSV). J. Gen. Virol..

[26] Xie J., Christiaens I., Yang B., Trus I., Devriendt B., Cui T., Wei R., Nauwynck H.J. (2018). Preferential use of Siglec-1 or Siglec-10 by type 1 and type 2 PRRSV strains to infect PK15(S1-CD163) and PK15(S10-CD163) cells. Vet. Res..

[27] Van Breedam W., Van Gorp H., Zhang J.Q., Crocker P.R., Delputte P.L., Nauwynck H.J. (2010). The M/GP(5) glycoprotein complex of porcine reproductive and respiratory syndrome virus binds the sialoadhesin receptor in a sialic acid-dependent manner. PLoS Pathog..

[28] Shanmukhappa K., Kim J.K., Kapil S. (2007). Role of CD151, A tetraspanin, in porcine reproductive and respiratory syndrome virus infection. Virol. J..

[29] Verheije M.H., Kroese M.V., van der Linden I.F., de Boer-Luijtze E.A., van Rijn P.A., Pol J.M., Meulenberg J.J., Steverink P.J. (2003). Safety and protective efficacy of porcine reproductive and respiratory syndrome recombinant virus vaccines in young pigs. Vaccine.

[30] Lee Y.J., Park C.K., Nam E., Kim S.H., Lee O.S., Lee du S., Lee C. (2010). Generation of a porcine alveolar macrophage cell line for the growth of porcine reproductive and respiratory syndrome virus. J. Virol. Methods.

[31] Wensvoort G., Terpstra C., Pol J.M., ter Laak E.A., Bloemraad M., de Kluyver E.P., Kragten C., van Buiten L., den Besten A., Wagenaar F. (1991). Mystery swine disease in The Netherlands: The isolation of Lelystad virus. Vet. Q..

[32] Reed L.J., Muench H. (1938). A Simple Method of Estimating Fifty Per Cent Endpoints12. Am. J. Epidemiol..

[33] Frydas I.S., Trus I., Kvisgaard L.K., Bonckaert C., Reddy V.R., Li Y., Larsen L.E., Nauwynck H.J. (2015). Different clinical, virological, serological and tissue tropism outcomes of two new and one old Belgian type 1 subtype 1 porcine reproductive and respiratory virus (PRRSV) isolates. Vet. Res..

[34] Frydas I.S., Nauwynck H.J. (2016). Replication characteristics of eight virulent and two attenuated genotype 1 and 2 porcine reproductive and respiratory syndrome virus (PRRSV) strains in nasal mucosa explants. Vet. Microbiol..

[35] Trus I., Frydas I.S., Reddy V.R., Bonckaert C., Li Y., Kvisgaard L.K., Larsen L.E., Nauwynck H.J. (2016). Immunity raised by recent European subtype 1 PRRSV strains allows better replication of East European subtype 3 PRRSV strain Lena than that raised by an older strain. Vet. Res..

[36] Kvisgaard L.K., Hjulsager C.K., Fahnoe U., Breum S.O., Ait-Ali T., Larsen L.E. (2013). A fast and robust method for full genome sequencing of Porcine Reproductive and Respiratory Syndrome Virus (PRRSV) Type 1 and Type 2. J. Virol. Methods.

[37] Kumar S., Stecher G., Tamura K. (2016). MEGA7: Molecular Evolutionary Genetics Analysis Version 7.0 for Bigger Datasets. Mol. Biol. Evol..

[38] Rappe J.C., Garcia-Nicolas O., Fluckiger F., Thur B., Hofmann M.A., Summerfield A., Ruggli N. (2016). Heterogeneous antigenic properties of the porcine reproductive and respiratory syndrome virus nucleocapsid. Vet. Res..

[39] Van Breedam W., Costers S., Vanhee M., Gagnon C.A., Rodriguez-Gomez I.M., Geldhof M., Verbeeck M., Van Doorsselaere J., Karniychuk U., Nauwynck H.J. (2011). Porcine reproductive and respiratory syndrome virus (PRRSV)-specific mAbs: Supporting diagnostics and providing new insights into the antigenic properties of the virus. Vet. Immunol. Immunopathol..

[40] Vanhee M., Delputte P.L., Delrue I., Geldhof M.F., Nauwynck H.J. (2009). Development of an experimental inactivated PRRSV vaccine that induces virus-neutralizing antibodies. Vet. Res..

[41] Costers S., Lefebvre D.J., Delputte P.L., Nauwynck H.J. (2008). Porcine reproductive and respiratory syndrome virus modulates apoptosis during replication in alveolar macrophages. Arch. Virol..

[42] Nauwynck H.J., Pensaert M.B. (1995). Effect of specific antibodies on the cell-associated spread of pseudorabies virus in monolayers of different cell types. Arch. Virol..

[43] Drake J.W., Holland J.J. (1999). Mutation rates among RNA viruses. Proc. Natl. Acad. Sci. USA.

[44] Elena S.F., Sanjuan R. (2005). Adaptive value of high mutation rates of RNA viruses: Separating causes from consequences. J. Virol..

[45] Klimstra W.B., Ryman K.D., Johnston R.E. (1998). Adaptation of sindbis virus to BHK cells selects for use of heparan sulfate as an attachment receptor. J. Virol..

[46] Lim B.L., Cao Y.C., Yu T., Mo C.W. (1999). Adaptation of very virulent infectious bursal disease virus to chicken embryonic fibroblasts by site-directed mutagenesis of residues 279 and 284 of viral coat protein VP2. J. Virol..

[47] Lohmann V., Korner F., Dobierzewska A., Bartenschlager R. (2001). Mutations in hepatitis C virus RNAs conferring cell culture adaptation. J. Virol..

[48] Zhang H.-L., Tang Y.-D., Liu C.-X., Xiang L.-R., Zhang W.-L., Leng C.-L., Wang Q., An T.-Q., Peng J.-M., Tian Z.-J. (2018). Adaptions of field PRRSVs in Marc-145 cells were determined by variations in the minor envelope proteins GP2a-GP3. Vet. Microbiol..

[49] Lu Z.H., Brown A., Wilson A.D., Calvert J.G., Balasch M., Fuentes-Utrilla P., Loecherbach J., Turner F., Talbot R., Archibald A.L. (2014). Genomic variation in macrophage-cultured European porcine reproductive and respiratory syndrome virus Olot/91 revealed using ultra-deep next generation sequencing. Virol. J..

[50] Fang Y., Rowland R.R., Roof M., Lunney J.K., Christopher-Hennings J., Nelson E.A. (2006). A full-length cDNA infectious clone of North American type 1 porcine reproductive and respiratory syndrome virus: Expression of green fluorescent protein in the Nsp2 region. J. Virol..

[51] Crooks G.E., Brenner S.E. (2005). An alternative model of amino acid replacement. Bioinformatics.

[52] Van Breedam W., Delputte P.L., Van Gorp H., Misinzo G., Vanderheijden N., Duan X., Nauwynck H.J. (2010). Porcine reproductive and respiratory syndrome virus entry into the porcine macrophage. J. Gen. Virol..

[53] Van Gorp H., Van Breedam W., Delputte P.L., Nauwynck H.J. (2009). The porcine reproductive and respiratory syndrome virus requires trafficking through CD163-positive early endosomes, but not late endosomes, for productive infection. Arch. Virol..

[54] Calvert J.G., Slade D.E., Shields S.L., Jolie R., Mannan R.M., Ankenbauer R.G., Welch S.K. (2007). CD163 expression confers susceptibility to porcine reproductive and respiratory syndrome viruses. J. Virol..

[55] Ma H., Jiang L., Qiao S., Zhi Y., Chen X.X., Yang Y., Huang X., Huang M., Li R., Zhang G.P. (2017). The Crystal Structure of the Fifth Scavenger Receptor Cysteine-Rich Domain of Porcine CD163 Reveals an Important Residue Involved in Porcine Reproductive and Respiratory Syndrome Virus Infection. J. Virol..

[56] Gallivan J.P., Dougherty D.A. (1999). Cation-pi interactions in structural biology. Proc. Natl. Acad. Sci. USA.

[57] Gorbalenya A.E., Enjuanes L., Ziebuhr J., Snijder E.J. (2006). Nidovirales: Evolving the largest RNA virus genome. Virus Res..

[58] Lauring A.S., Andino R. (2010). Quasispecies Theory and the Behavior of RNA Viruses. PLoS Pathog..

[59] Lauring A.S., Frydman J., Andino R. (2013). The role of mutational robustness in RNA virus evolution. Nat. Rev. Microbiol..

[60] Goldberg T.L., Lowe J.F., Milburn S.M., Firkins L.D. (2003). Quasispecies variation of porcine reproductive and respiratory syndrome virus during natural infection. Virology.

